# Non-Conjugated Copoly(Arylene Ether Ketone) for the Current-Collecting System of a Solar Cell with Indium Tin Oxide Electrode

**DOI:** 10.3390/polym15040928

**Published:** 2023-02-13

**Authors:** Alexey N. Lachinov, Danfis D. Karamov, Azat F. Galiev, Sergey N. Salazkin, Vera V. Shaposhnikova, Tatiana N. Kost, Alla B. Chebotareva

**Affiliations:** 1 Skobeltsyn Institute of Nuclear Physics, Lomonosov Moscow State University, 119991 Moscow, Russia; 2 Institute of Molecule and Crystal Physics–Subdivision of the Ufa Federal Research Centre of the Russian Academy of Sciences, 450075 Ufa, Russia; 3 Nesmeyanov Institute of Organoelement Compounds of the Russian Academy of Sciences, 119334 Moscow, Russia

**Keywords:** copoly(arylene ether ketone), non-conjugated polymer, indium tin oxide, potential barrier, charge carrier concentration, charge carrier mobility, solar cell

## Abstract

The mechanism of charge carrier transport in the indium tin oxide (ITO)/polymer/Cu structure is studied, where thin films of copoly(arylene ether ketone) with cardo fluorene moieties are used. This copoly(arylene ether ketone) is non-conjugated polymer which has the properties of electronic switching from the insulating to the highly conductive state. The dependence on the polymer film thickness of such parameters as the potential barrier at the ITO/polymer interface, the concentration of charge carriers, and their mobility in the polymer is studied for the first time. The study of this system is of interest due to the proven potential of using the synthesized polymer in the contact system of a silicon solar cell with an ITO top layer. The parameters of charge carriers and ITO/polymer barrier are evaluated based on the analysis of current–voltage characteristics of ITO/polymer/Cu structure within the injection current models and the Schottky model. The thickness of the polymer layer varies from 50 nm to 2.1 µm. The concentration of intrinsic charge carriers increases when decreasing the polymer film thickness. The charge carrier mobility depends irregularly on the thickness, showing a maximum of 9.3 × 10^−4^ cm^2^/V s at 210 nm and a minimum of 4.7 × 10^−11^ cm^2^/V s at 50 nm. The conductivity of polymer films first increases with a decrease in thickness from 2.1 µm to 210 nm, but then begins to decrease upon transition to the globular structure of the films at smaller thicknesses. The dependence of the barrier height on polymer thickness has a minimum of 0.28 eV for films 100–210 nm thick. The influence of the supramolecular structure and surface charge field of thin polymer films on the transport of charge carriers is discussed.

## 1. Introduction

Currently, photovoltaics is actively developing new approaches to the formation of current-collecting contacts in solar cells with an upper layer of transparent conducting oxides (TCO) using economical low-temperature processes with a reduction in silver usage [1]. This is relevant, since the rise in prices for silver slows down the decline in the price of solar Watt peak, and the limited world reserves of silver are an obstacle to the development of silicon photovoltaics to the terawatt scale [2].

The TCO layers are an almost unavoidable component in most types of solar cells; this primarily applies to highly efficient silicon solar cells based on the TCO/α-Si:H/c-Si heterojunction (SHJ) [3,4]. These SHJ solar cells impose certain limitations on the temperature of current-collecting system formations because of the possible degradation of the electrical and optical properties of the TCO and α-Si:H layers and interfaces [5,6]. At present, SHJ solar cells are produced with standard screen-printed contact fingers from expensive silver-containing pastes with low curing temperatures (typically ≤ 200 °C) [7]. The best bulk resistivities of modern pastes are in the range of 5 μΩ cm [8], but this is three times greater than the bulk resistivity of copper. Silver inkjet printing and FlexTrail printing are novel printing technologies; both use the same Ag nanoparticle ink and have a curing temperature of 200 °C [9]. FlexTrail printing allows researchers to reduce the contact finger width to 16 μm compared to 75 μm for inkjet printing and screen printing. The amount of silver ink consumed during FlexTrail printing is extremely low, but the resistivity of the Ag nanoparticle ink is higher than that of the best silver pastes, namely 10 μΩ cm, and contact resistivity to ITO is also high, at 5 mΩ cm^2^ [9].To entirely suppress the need for silver, copper plating has also been reported as an alternative approach [10]. However, the adhesion of copper may be problematic if direct electrodeposition on the TCO is performed. A rather complex and expensive process is needed to produce plated fingers [10].

Over the past decade, leading centers in the field of silicon photovoltaics have been actively conducting research aimed at promoting a new, progressive technology for multi-wire metallization and the interconnection of solar cells in a module based on the use of copper wires coated with low-temperature indium-containing solder [4]. This technology uses many wires in the place of busbars, and the copper wires are “soldered” during the low-temperature lamination process to the fingers (printed or plated) and to the TCO layer, e.g., in the case of the TCO/α-Si:H/c-Si heterojunction cells. With the use of multi-wire configurations, the demand on finger conductance is further decreased, and it considerably reduces silver consumption (by 80%), while boosting efficiency due to significantly decreased ohmic losses [11].

However, multi-wire design suffers from the disadvantage of using an expensive In-containing solder (~10 mg/Watt peak) because indium, like silver, is expensive and is scarce. It is for this reason that In-containing solder is now replaced with Bi:Sn low temperature solder to reduce the cost per Watt peak [12].

Another approach proposed earlier by the authors is the use of multi-wire metallization, when a current-collecting grid of multiple copper wires is glued directly to the TCO surface using conductive polymeric materials [13]. This contact system is Ag-free, since it does not require the formation of any contact fingers, except for wires. To create such a contact, transparent polymeric materials with metallic conductivity and good adhesion to TCO and metal surfaces can be used. However, it is well known that the use of traditional conjugated polymers is difficult due to their poor resistance to aggressive environment and solar radiation. As a rule, these factors lead to the degradation of the molecular structure and the deterioration of the electrically conductive properties [14]. Non-conjugated polymeric materials have a significantly higher resistance to aggressive environment, temperature, and radiation. Non-conjugated polymers have a large difference between the energies of the highest occupied orbital (HOMO) and the lowest vacant orbital (LUMO) and, therefore, are poor current conductors. However, in thin films of some non-conjugated polymers of the polyarylene class, conditions were found [15] under which high conductivity of a metallic type can arise in them [16]. It should be emphasized that high conductivity was achieved without the use of doping or the introduction of any electrically conductive fillers into the polymer matrix. As a result, it was possible to achieve a unique combination of high metallic conductivity and optical transparency in the visible and ultraviolet regions of the spectrum, which makes these polymers promising as transparent electrically conductive molecularly homogeneous materials. Most often, when creating a contact in a metal (semiconductor)/polymer structure, a polymer film was created by deposition from a solution. In the technology of solar cell processing, the additional use of solutions may be undesirable. Therefore, it is important in the technology discussed to use thermoplastic non-conjugated materials as electrically conductive adhesives to form current-collecting wire contacts to solar cells.

It has previously been reported that thermoplastic cardo copoly(arylene ether ketones) (co-PAEK) containing fluorene moieties can be used for this. The priority of the synthesis and study of the electrical properties of copoly(arylene ether ketone) with cardo fluorene moieties belongs to the authors of the article. Despite extensive research in the field of cardo aromatic polyethers carried out by scientists from different countries, there is still no information on the study of the electrical properties of these polymers by other groups. These polymers provided an electrical contact with TCO and metal with low resistivity (less than 1 mΩ cm^2^) [17]. The use of these polymers made it possible to obtain good adhesion even with round copper wires without the use of low-temperature In: Sn solder. It should be noted that the mechanisms of charge carrier transport in the TCO/co-PAEK/metal structure have not been sufficiently studied, which complicates the further development of this technology. Furthermore, the optimal thickness of the polymer film, at which the minimum resistance of the TCO/polymer/metal contact will be observed, has not yet been determined. In this regard, the aim of this work was to study the mechanisms of charge carrier transport in the ITO (In_2_O_3_:Sn)/co-PAEK/Cu system depending on the thickness of the polymer film. The most common top electrode material in silicon solar cells is ITO. The range of polymer film thicknesses (up to 2 μm) corresponds to the distances between the metal wires and the surface of the solar cell after contact formation [18].

## 2. Materials and Methods

### 2.1. Polymer

**Figure 1:**
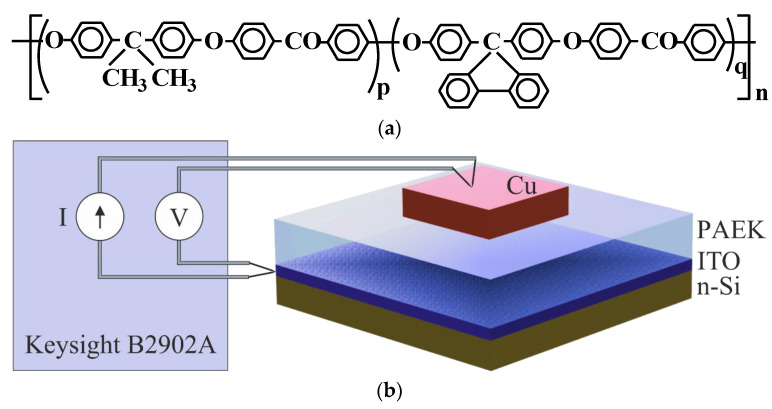
(**a**) Structural formula of co-PAEK, where p/q = 0.90/0.10; (**b**) block diagram of measurements and schematic structure of the Si/ITO/co-PAEK/Cu sample.

Figure 1a shows the chemical structure of the synthesized co-PAEK with a fluorene cardo group *q* and isopropylidene auxiliary group *p*. The content of fluorene-containing units, *q*, was 10 mol%. The co-PAEK was synthesized according to procedures reported in [19]. The polycondensation conditions were as follows. The concentration of each monomer was 0.5 mol per 1 L of the solvent and 30% excess K_2_CO_3_. The procedure used in the synthesis of co-PAEK based on 4,4′-difluorobenzophenone and a mixture of 2,2-bis(4′-hydroxyphenyl)propane and 9,9-bis(4′-hydroxyphenyl)fluorene is outlined below.

4,4′-Difluorobenzophenone, 2,2-bis(4′-hydroxyphenyl)propane, 9,9-bis(4′-hydroxyphenyl)fluorene, 4-fluorobenzophenone, *N*,*N*-dimethylacetamide, chlorobenzene, potassium carbonate, and other chemicals, all of analytical grade, were purchased from Acros Organics Co. Ltd. Anhydrous K_2_CO_3_ was thoroughly triturated and dried at 130 °C for 3 h before use.

A four-neck flask equipped with a stirrer, thermometer, inlet for feeding argon, and setting for the azeotropic distillation of water was purged with argon and charged with 4,4′-difluorobenzophenone (0.1 mol), 2,2-bis(4′-hydroxyphenyl)propane (0.09 mol), 9,9-bis(4′-hydroxyphenyl)fluorene (0.01 mol), 4-fluorobenzophenone (0.002 mol), K_2_CO_3_ (0.13 mol) (which was thoroughly ground and calcined before use), *N*,*N*-dimethylacetamide (200 mL), and chlorobenzene (100 mL). The flask placed into an oil bath whose temperature was gradually (within ca. 0.5 h) increased to 185 °C. After a chlorobenzene–water azeotropic mixture was distilled off, the reaction was conducted for 7 h. When the synthesis was complete, the reaction mixture was cooled, ridded from salts by filtration and washed with water. The polymer was obtained in the form of a film. To this end, its chloroform solution was evaporated at 25 °C and dried. Initially, the temperature was gradually increased from 60 to 140 °C for 18 h, and then drying was continued at 160 °C for 25 h. The polymer yield was 99%.

The reduced viscosity (*η*_red_) determined for a solution of the polymer in chloroform (0.5 g/100 mL) at 25 °C using an Ubbelohde viscometer with a capillary diameter of 0.6 mm was as follows: *η*_red_ = 0.38 dL g^−1^.

Average molecular weights and molecular weight distribution were determined by gel permeation chromatography on an Agilent 1100 instrument with a UV–VIS detector and two Ultrastyragel linear columns in chloroform, T = 25 °C. Universal calibration was carried out using narrowly dispersed PS standards (Merck and Waters), using the following coefficients of the Mark–Houwink equation: K = 3.90 × 10^−4^ and α = 0.72, determined for PAEK based on 4,4′-difluorobenzophenone and 2,2-bis(4′-hydroxyphenyl)propane [20]. The polymer has the following average molecular weights: number-average molecular weight M_n_ = 5620 Da; weight-average molecular weight M_w_ = 20,324 Da. Polydispersity (PD) was calculated as PD = M_w_/M_n_ = 3.62. The co-PAEK glass transition temperature (*T*_g_) was determined by thermo-mechanical analysis (TMA), *T*_g_ = 166 °C. The TMA was carried out on a TA Instruments Q400 TMA under a load of 0.1 MPa at a rate of 1.5 °C/min in compression mode. The samples had the form of pressed films about 300 μm in thickness.

The co-PAEK was highly soluble in a wide range of solvents (dichloromethane, chloroform, sym-tetrachloroethane, tetrahydrofurane, dioxane, cyclohexanone, *m*-cresol, *N*,*N*-dimethylformamide*, N*,*N*-dimethylacetamide, etc.); upon formation from a solution, the polymers formed transparent (in the wavelength range of 400–1200 nm) strong films (the tensile strength was 75–82 MPa).

### 2.2. Si/ITO/co-PAEK/Cu Test Samples for I-V Measurements

The samples for studying the current–voltage (*I-V*) characteristics had a Si/ITO/co-PAEK/Cu multilayer structure (Figure 1b). An ITO layer about 100 nm thick with a resistivity of 7.6 mΩ cm was deposited on the surface of polished n-type silicon (with a resistivity of 18 Ω cm) by ultrasonic spray pyrolysis [21]. The ITO/Si structure simulated the silicon solar cell surface.

The polymer film was deposited on the surface of the ITO layer by spin-coating from a polymer solution in cyclohexanone at 1500 rpm for 1 min. The thickness of polymer films was set by varying the concentration of the solutions in the range from 1.25 to 15 wt.%.

After applying the polymer film, the sample was subjected to two-stage drying, with 1 h at a temperature of 25 °C, then 10 h at a temperature of 150 °C. The copper electrode with an area of 5 × 5 mm^2^ and a thickness of 2 µm was deposited by thermal diffusion in vacuum.

The surface morphology was analyzed, and the thickness of the polymer films was measured by atomic force microscopy (AFM) using an SMM-2000T microscope (JSC Proton Plant, Zelenograd, Russia) in the contact mode. To determine the film thickness, a recess was created in them up to the substrate surface using a copper micro-cutter. The recess wall is a step with a height equal to the required thickness. The true morphology of the surface section with a step is reproduced in the form of an AFM image, on which it becomes possible to construct a surface section profile. The film thicknesses were varied in a range from 50 nm for 1.25 wt.% to 2.1 µm for 15 wt.%.

To obtain numerical information about the roughness, a series of images with the same dimensions was taken from different parts of the surface. For each image, the root-mean-square roughness (*R*_q_) was calculated, which gives the root-mean-square average of height deviations taken from the mean data plane within a given area. Parameter *R*_q_, extracted from the AFM measurements, was obtained for the mapping areas of 7.5 × 7.5 μm^2^. This parameter was used to evaluate the surface quality of the obtained films.

The electrical conductivity of the experimental structure was studied by measuring the I-V characteristics using a Keysight B2902A precision parametric analyzer at an MPI ETS50 probe station in the temperature range of 25–125 °C. The temperature interval was chosen, taking into account the operating conditions of solar cells. At least five samples of each thickness were used during the measurements. The reproducibility of the results was satisfactory, and the measurement error did not exceed 15%.

## 3. Results and Discussion

### 3.1. Morphology Analysis of Co-PAEK Films

**Figure 2:**
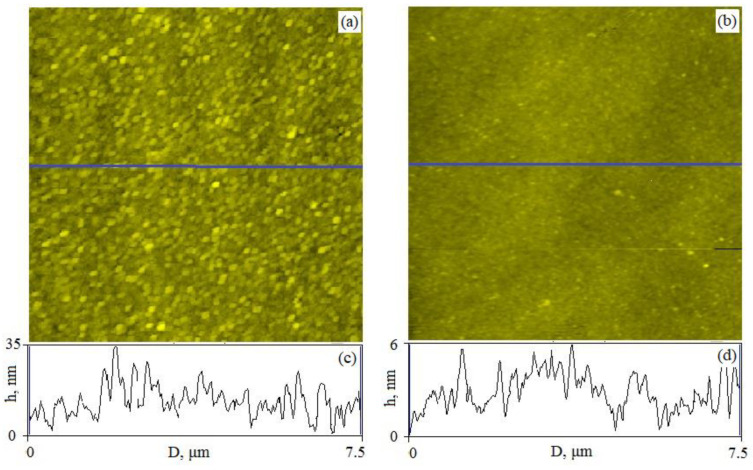
AFM images of the samples surface. The dimensions of the scan areas are 7.5 × 7.5 µm^2^. (**a**) Image of the ITO surface (*R*_q_ = 5.0 nm); (**b**) image of the surface of a 50 nm thick co-PAEK polymer film formed on the ITO surface (*R*_q_ = 1.4 nm); (**c**,**d**) surface profiles measured along the measurement lines shown in figures. (**a**,**b**) Solid lines; (**c**) ITO surface profile; (**d**) polymer film surface profile.

The results of the estimates are presented in Table 1. With an increase in the thickness of the polymer film, the *R*_q_ value decreased from 1.4 to 1.0 nm (Table 1). Taking into account the granular nature of the ITO layer (*R*_q_~5.0 nm) (see Figure 2), it can be argued that during the production of films, the polymer material fills all the irregularities of the substrate surface. The estimates obtained indicate a low degree of surface roughness for polymer films and their high film-forming properties.

Figure 2 shows the results of AFM control of the morphology and surface roughness of the samples.

### 3.2. Analysis of I-V Characteristics of the ITO/co-PAEK/Cu Structure

**Figure 3:**
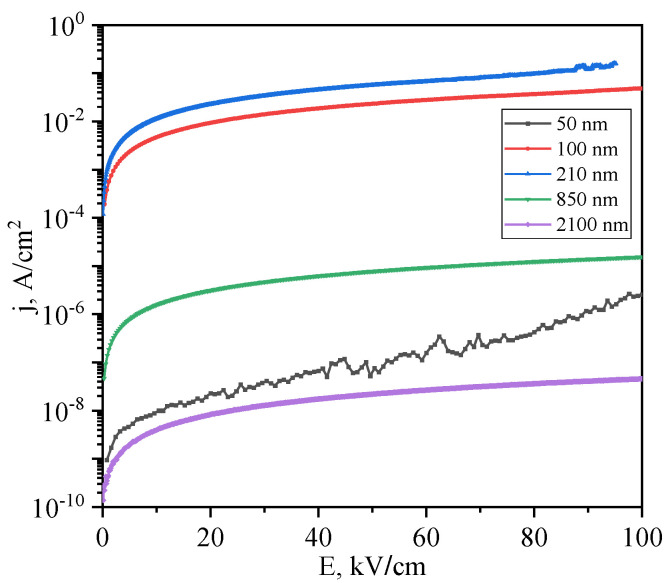
Current–voltage characteristics of ITO/co-PAEK/Cu heterostructures, measured at room temperature, for different co-PAEK thicknesses.

Figure 3 shows the *I*-*V* characteristics of the ITO/co-PAEK/Cu heterostructures measured at different thicknesses of the polymer film. The *I*-*V* characteristics are presented at one polarity of the applied field due to the fact that the characteristics turned out to be symmetrical with respect to changing the direction of current flow. This may indicate that the potential barriers on both sides of the polymer film are the same. When using ITO as an electrode, such symmetry of the *I*-*V* curves is often observed. This is explained by the fact that ITO is a degenerate semiconductor with a wide range of electronic states in the band gap. This means that in the spectrum of electronic states of ITO, the injection level and the Fermi level of copper can have practically the same energy.

The current–voltage characteristics presented in Figure 3 show a non-trivial dependence of the film resistance on their thickness. As the thickness decreases from 2100 nm to 210 nm, the resistance of the samples decreases, and the current increases at the same voltage. For example, at an electric field strength of 20 kV/cm, the resistivity drops from 2.4 × 10^12^ Ω·cm to 8.8 × 10^5^ Ω·cm. A further decrease in thickness to 50 nm leads to an increase in resistance from 2.1∙10^6^ Ω·cm at 100 nm to 8.7 × 10^11^ Ω·cm at 50 nm.

An analysis of the shape of the current–voltage characteristics showed that, regardless of the thickness, the *I*-*V* response is characterized by a nonlinear dependence of the current on the applied voltage. This dependence can be described by a power law of the form, as follows:

(1) $$I\propto kU^{n}$$

where *I* is the current flowing through the film sample, *U* is the potential difference, *k* is the proportionality factor, which may depend on the concentration and mobility of charge carriers, thickness, and other parameters, *n* is the exponent, which is greater than unity and, depending on its value, can contain information about the energy distribution of electronic states in the tails of the energy bands. Such current dependences on the applied voltage are often observed in heterostructures containing metal/polymer contacts [22].

At low voltages, this dependence is close to linear, that is, to Ohm’s law. As the voltage increases above a certain threshold value *U*_thr_, the exponent becomes greater than one. The *I*-*V* curve goes into the so-called super linear mode. Such a behavior of the current *j* as a function of the applied voltage *U* in a metal/polymer/semiconductor structure is often interpreted in terms of the Mott–Gurney injection model [23].

According to this model, the linear part of the *I*-*V* characteristic is due to intrinsic charge carriers, and is described by the following relationship [23]:

(2) $$j_{1}=\frac{en_{0}\mu U}{L}$$

where e is the electron charge, *n*_0_ is the concentration of intrinsic charge carriers, *µ* is the mobility of charge carriers, and *L* is the thickness of the polymer film. The nonlinear part of the *I*-*V* characteristic is related to the charge carriers injected from the electrode. In this case, it is described by another expression, as follows [23]:

(3) $$j_{2}=\frac{\varepsilon \varepsilon_{0}\mu U^{2}}{L^{3}}$$

where *ε* is the polymer permittivity, *ε* = 3.4; ε_0_ is the dielectric constant, and *ε*_0_ = 8.85 × 10^−12^ F·m^−1^.

According to the model [24], the equality of currents *j_1_* and *j_2_* arises at a certain voltage *U*_thr_, at which the current of injected charge carriers begins to exceed the current of intrinsic charge carriers. Solving a system of two equations with two unknowns (*n*_0_ and *µ*), it is easy to determine their values. It should be noted that in this case the concentration of equilibrium intrinsic charge carriers and the minimum charge carrier mobility is estimated.

Thus, one can obtain for the concentration of charge carriers, as follows [24]:

(4) $$n_{0}=\frac{\varepsilon \varepsilon_{0}U_{thr}}{eL^{2}}$$

for charge carrier mobility, the following can be obtained:

(5) $$\mu =\frac{jL^{3}}{\varepsilon \varepsilon_{0}U_{thr}^{2}}$$

Where *U*_thr_ is defined on the *I-V* characteristic plotted in logarithmic coordinates as the voltage value at the point of intersection of the linear and super-linear approximations.

The results of estimates of the concentration and mobility of charge carriers for polymer films of different thicknesses are presented in Table 2.

The results obtained showed that the best correlation with the film thickness is observed for the voltage *U*_thr_ on the *I*-*V* characteristics. This parameter decreases when decreasing the thickness, from 3.1 V at 2100 nm to 0.2 V at 50 nm. At the same time, the concentration of intrinsic charge carriers increases by two orders of magnitude with decreasing sample thickness. However, the charge carrier mobility depends irregularly on the thickness, showing a maximum of 9.3 × 10^−4^ cm^2^/V s at 210 nm. In this case, an extremely low mobility was obtained at a film thickness of 50 nm. Previously, the mobility in polydiphenylenephthalide films was measured by the time-of-flight method, which showed that at a film thickness of ~500–800 nm, the mobility is ~10^−6^ cm^2^/V s [25]. These values are close to those presented in the table for a thickness of 850 nm. Comparing the results of the estimates of the parameters of charge carriers with changes in the conductivity of polymer films, one can conclude that the main factor affecting the conductivity with a change in film thickness is the change in the charge carrier mobility.

However, there is another important factor that can affect the injection of charge carriers in the metal/polymer/semiconductor structure, that is, the height of the potential barrier *φ_B_* at the metal (semiconductor)/polymer interface. The value of *φ_B_* can be estimated using the Richardson–Dushman equation [26]. From this equation it is easy to obtain the potential barrier height (6), as follows:

(6) $$\varphi_{B}=\frac{kT}{e}\ln\left(\frac{SA^{**}T^{2}}{I_{S}}\right)$$

where *T* is the temperature, *k* is the Boltzmann constant, *e* is the electron charge, *S* is the contact area, A** is the Richardson constant, A** ≈ 110 [A cm^−2^K^−2^], and *I_S_* is the saturation current. Experimental data make it possible to determine *I*_S_ as the point of intersection of a straight line passing through the *I-V* characteristic saturation region with the voltage *axis* (*U* = 0) on a graph plotted in semilogarithmic coordinates (ln(*I*) − *U*).

The results of the corresponding calculation of *φ_B_* are presented in Table 2. It is interesting that the change in barrier heights for samples with polymer films of different thicknesses correlates with the change in the conductivity of these samples. The dependence of the barrier heights on the thickness has an extreme character, with a minimum for films which are100 and 210 nm thick. It is with these films that the conductivity maximum is observed.

The most probable explanation of the obtained thickness dependences can be obtained if two factors are taken into account. The first factor is the change in the supramolecular structure of polymer films when their thickness changes. This factor directly affects the processes of charge carrier scattering. The second factor is an increase in the role of surface states (surface charge fields) on injection processes with a decrease in the thickness of polymer films.

Let us consider the first factor. It is known that the relative strength of polymer–polymer and polymer–solvent interactions determines the initial state of polymers in solution [27]. Depending on the molecular structure and polymer–solvent interaction, different conformations of polymers and, accordingly, the degree of their aggregation, are possible. The state of the polymer in solution has a decisive influence on chain conformation, packing, and charge transfer in the solid state. This idea is the basis of the strategy of “control of the supramolecular structure in the solution state” to control the electrophysical and optical properties of thin films [28,29]. In this case, the strength of the polymer–solvent interaction can be controlled by changing the concentration of the polymer in the solution or the temperature of the solution [30]. Films were made by centrifugation from a polymer solution. Previously it was found that, depending on the concentration of polymer solutions, not only the film thickness but also the nature of the supramolecular ordering in the film changes [31]. Large aggregates in the form of discs with a diameter of 20 to 150 nm are formed in films made from solutions with a concentration above 2 wt.%. Globules appear in films made from solutions of lower concentrations.

In the present work, films less than 210 nm thick were obtained from solutions with concentrations below 5 wt.%. Furthermore, it is in the region of these thicknesses that the character of charge transfer changes. In particular, as the thickness decreases, the resistance of polymer films begins to increase. Apparently, this is due to the fact that the globular structure of the films implies an increase in the quantity of interglobular charge carrier hopping and the associated additional scattering. This leads to an increase in the resistance of the films. Previously, it was reported [32] that the transition from a globular structure to aggregates leads to a sharp change in the activation energy of trap centers. This is manifested in a significant decrease in the effective charge carrier mobility in the region of these thicknesses (see Table 2).

In the case of 50 nm-thick film, additional factors arise that prevent the transport of charge carriers. The most significant factor is the surface charge field, which is directed opposite to the external field, partially compensating for its effect. The presence of a surface charge leads to an increase in the height of the potential barrier and a decrease in the injection of charge carriers from the electrode into the polymer film. This conclusion is confirmed by the estimate of the potential barrier height presented in Table 2 for the 50 nm thick film, as well as by the anomalously low value of the charge carrier mobility.

**Figure 4:**
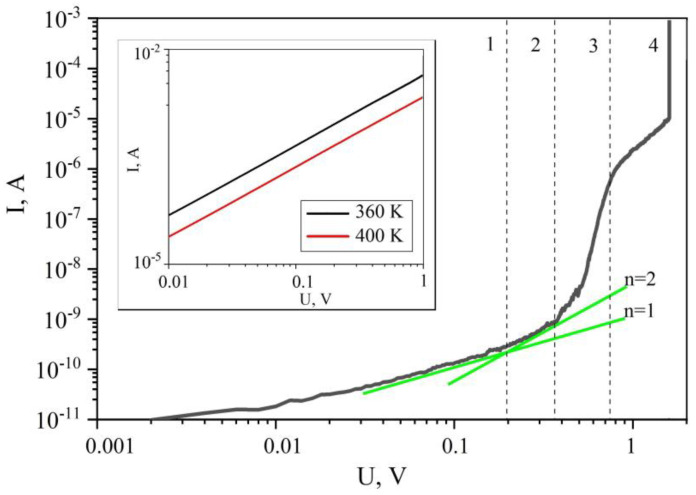
Current–voltage characteristic of the ITO/co-PAEK/Cu structure in logarithmic coordinates, measured at a temperature of 300 K. The thickness of the polymer film is 50 nm. The vertical dashed lines mark the regions (1,2,3,4) on the *I*-*V* curve, which characterize different stages of the injection process. The inset shows two *I*-*V* curves of the sample in a highly conductive state, measured at temperatures of 360 K and 400 K, respectively.

The answer to the question about the change in the conductivity of a thin film under the condition of compensation of the field of the built-in charge is of interest. The answer to this question is presented in Figure 4. This is the *I*-*V* characteristic of a thin film sample, measured in a range of fields larger than those shown in Figure 3. Compensation of the internal field occurs due to a change in the concentration of the space charge during the injection of electrons from the electrodes. At low voltages (region 1 in Figure 4), charge carriers are transported due to their own current carriers. In this regard, the CVC has an ohmic dependence, which corresponds to n = 1 (see Equation (1)). In region 2, the concentration of injected charge carriers begins to exceed the concentration of intrinsic charge carriers, and, therefore, “n” increases to 2. In this region, the field of the built-in charge is compensated by the field of the injected charges. In this regard, the quasi-Fermi level falls into the energy interval in which the electronic trap states are localized. As a result, there is a sharp increase in the density of the flowing current by three orders of magnitude. This region is characterized by the exponent n >> 2. In the injection model, this region is called the limiting trap filling region. The process of filling the traps is accompanied by an increase in the density of charge transfer centers. At a certain concentration, the electronic functions of such centers overlap, and a narrow charge transfer zone appears. This leads to the effect of electronic switching of conductivity, as a result of which the sample passes into a metal-like state (Figure 4, vertical section in region 4). Such a scheme for the transition to a metal-like state has been experimentally confirmed [33]. The metal-like nature of the conductivity that occurs after the electronic transition is confirmed by the results of measurements of the *I*-*V* characteristics at different temperatures (inset in Figure 4). It is clearly seen that an increase in the sample temperature leads to a decrease in the flowing current. Thus, it has been found that the decrease in conductivity in thin polymer films is associated with the influence of the built-in charge field. Compensation of this field leads to electronic switching of the polymer film into a metal-like state.

## 4. Conclusions

The copoly(arylene ether ketone) with a fluorene cardo group (10 mol%), having low reduced viscosity (0.38 dL g^−1^), was synthesized. The features of charge carrier transport in the ITO/copoly(arylene ether ketone)/Cu structure were studied for polymer film thicknesses from 50 nm to 2.1 µm. A low degree of surface roughness of polymer films (≤1.4 nm) and their high film-forming properties were estimated by atomic force microscopy.

The concentration of intrinsic charge carriers increases by two orders of magnitude–from 1.4 × 10^14^ to 1.8 × 10^16^ cm^−3^ when decreasing the polymer film thickness from 2.1 µm to 100 nm. However, the charge carrier mobility depends irregularly on the thickness, showing a maximum of 9.3 × 10^−4^ cm^2^/V s at 210 nm and a minimum of 4.7 × 10^−11^ cm^2^/V s at 50 nm. The conductivity of polymer films first increases with a decrease in thickness from 2.1 µm to 210 nm, but then begins to decrease upon transition to the globular structure of the films at smaller thicknesses. The decrease in conductivity is enhanced by increasing the influence of the surface charge field for a polymer film thickness of 50 nm. The dependence of the barrier heights on polymer thickness has a minimum of 0.28 eV for 100 and 210 nm thick films and maximum of 0.62 eV for the film which was 2.1 µm thick. In the case of 50 nm-thick film, the presence of a surface charge leads to an increase in the height of the potential barrier and a decrease in the injection of charge carriers from the electrode into the polymer film.

It has been found that the transport of charge carriers in thin films of copoly(arylene ether ketones) in the ITO/polymer/Cu structure depends on the supramolecular structure of polymer films. In the manufacture of films by spin-coating from solution, the molecular aggregates formed in the solution determine the supramolecular ordering in the film. Therefore, a change in the supramolecular ordering when exposed to highly dilute solutions and the formation of globules promotes the formation of films of a different structure.

In this regard, the conductivity of polymer films initially increases with a decrease in thickness, but then begins to decrease upon transition to a globular structure. The decrease in conductivity is enhanced by increasing the influence of the surface charge field with a decrease in polymer film thickness to 50 nm. This is indirectly confirmed by an increase in the intrinsic charge carrier concentration. However, a more significant decrease in the charge carrier mobility and an increase in the potential barrier led to a decrease in the conductivity of the structure. In the case of 50 nm-thick film, the presence of a surface charge leads to an increase in the height of the potential barrier and a decrease in the injection of charge carriers from the electrode into the polymer film.

## Figures and Tables

**Table 1:** Results of the evaluation of the thickness *L* and roughness *R*_q_ of polymer films obtained by spin-coating from co-PAEK solutions of various concentrations using AFM.

#	wt. [%]	*L* [nm] (±5%)	*R*_q_ [nm] (±5%)
1	1.25	50	1.4
2	2.5	100	1.0
3	5	210	1.0
4	10	850	1.1
5	15	2100	0.9

**Table 2:** Parameters of co-PAEK films depending on the thickness *L*. Here, *I*_S_ is the saturation current, *U*_thr_ is the threshold voltage for the transition to the super-linear regime, *µ* is the charge carrier mobility, *n*_0_ is the charge carriers concentration, and *φ*_B_ is the potential barrier height at the ITO/polymer interface.

*L*. nm	*I*_S_, A	*U*_thr_, V	*µ*, cm^2^/V·s	*n*_0_, cm^−3^	*φ_B_*, eV
50	2.8 × 10^−10^	0.2	4.7 × 10^−11^	1.6 × 10^16^	0.51
100	2.4 × 10^−3^	0.91	1.5 × 10^−4^	1.8 × 10^16^	0.28
210	4.6 × 10^−3^	1.55	9.3 × 10^−4^	7.0 × 10^15^	0.28
850	3.9 × 10^−8^	2.15	2.7 × 10^−7^	5.9 × 10^14^	0.52
2100	4.4 × 10^−10^	3.1	2.2 × 10^−8^	1.4 × 10^14^	0.62

## Data Availability

Not applicable.

## Nomenclature

ITO—indium tin oxide	*e*—electron charge
TCO—transparent conductive oxide	*n*_0_—concentration of charge carriers
HOMO—the highest occupied orbital	*µ*—mobility of charge carriers
LUMO—the lowest vacant orbital	*ε*—polymer permittivity
co-PAEK—copoly(arylene ether ketone)	*ε*_0_—dielectric constant
TMA—thermo-mechanical analysis	*I_S_*—saturation current
AFM—atomic force microscopy	*U*_thr_—threshold voltage for the transition to the super-linear regime
M_n_—number-average molecular weight	*φ_B_*—potential barrier height
M_w_—weight-average molecular weight	*T*—temperature
PD—polydispersity	*k*—Boltzmann constant
*η*_red_—reduced viscosity	*S*—contact area
*R*_q_—root-mean-square roughness	*A***—Richardson constant
*L*—thickness of the polymer film	

## References

[1] Descoeudres A., Allebé C., Badel N., Barraud L., Champliaud J., Christmann G., Debrot F., Faes A., Geissbühler J., Horzel J. (2018). Low-temperature processes for passivation and metallization of high efficiency crystalline silicon solar cells. Sol. Energy.

[2] Zhang Y., Kim M., Wang L., Verlinden P., Hallam B. (2021). Design considerations for multi-terawatt scale manufacturing of existing and future photovoltaic technologies: Challenges and opportunities related to silver, indium and bismuth consumption. Energy Environ. Sci..

[3] Taguchi M., Yano A., Tohoda S., Matsuyama K., Nakamura Y., Nishiwaki T., Fujita K., Maruyama E. (2014). 24.7% record efficiency HIT solar cell on thin silicon wafer. IEEE J. Photovolt..

[4] Liu Y., Li Y., Wu Y., Yang G., Mazzarella L., Procel-Moya P., Tamboli A.C., Weber K., Boccard M., Isabella O. (2020). High-Efficiency Silicon Heterojunction Solar Cells: Materials, Devices and Applications. Mater. Sci. Eng. R Rep..

[5] De Wolf S., Kondo M. (2009). Nature of doped a-Si:H/c-Si interface recombination. J. Appl. Phys..

[6] Haschke J., Dupré O., Boccard M., Ballif C. (2018). Silicon heterojunction solar cells: Recent technological development and practical aspects–from lab to industry. Sol. Energy Mater. Sol. Cells.

[7] Kanneboina V. (2022). Detailed review on c-Si/a-Si:H heterojunction solar cells in perspective of experimental and simulation. Microelectron. Eng..

[8] Geissbühler J., Faes A., Lachowicz A., Ballif C., Despeisse M. (2017). Metallization techniques and interconnection schemes for high-efficiency silicon heterojunction photovoltaics. Photovolt. Int..

[9] Schube J., Fellmeth T., Jahn M., Keding R., Glunz S.W. (2019). Inkjet- and FlexTrail-Printing with Low Silver Consumption for Silicon Heterojunction Solar Cells. Phys. Status Solidi RRL.

[10] Yu J., Li J., Zhao Y., Lambertz A., Chen T., Duan W., Liu W., Yang X., Huang Y., Ding K. (2021). Copper metallization of electrodes for silicon heterojunction solar cells: Process, reliability and challenges. Sol. Energy Mater. Sol. Cells.

[11] Papet P., Andreetta L., Lachenal D., Wahli G., Meixenberger J., Legradic B., Frammelsberger W., Batzner D., Strahm B., Yao Y. (2015). New cell metallization patterns for heterojunction solar cells interconnected by the smart wire connection technology. Energy Procedia.

[12] Spinella L., Bosco N. (2021). Thermomechanical fatigue resistance of low temperature solder for multiwire interconnects in photovoltaic modules. Sol. Energy Mater. Sol. Cells.

[13] Chebotareva A.B., Untila G.G., Kost T.N., Stepanov A.S., Salazkin S.N., Shaposhnikova V.V. (2017). Transparent conductive polymers for laminated multi-wire metallization of bifacial concentrator crystalline silicon solar cells with TCO layers. Sol. Energy Mater. Sol. Cells.

[14] Schultheiss A., Gueye M., Carella A., Benayad A., Pouget S., Faure-Vincent J., Demadrille R., Revaux A., Simonato J.-P. (2020). Insight into the Degradation Mechanisms of Highly Conductive Poly(3,4-ethylenedioxythiophene) Thin Films. ACS Appl. Polym. Mater.

[15] Lachinov A.N., Vorob’eva N.V. (2006). Electronics of thin layers of wide-band polymers. Phys. Uspekhi.

[16] Galiev A.F., Lachinov A.N., Kornilov V.M., Gadiev R.M. (2020). Temperature Dependence of the Resistance of Thin Poly(Diphenylene Phthalide) Films. Bull. Russ. Acad. Sci. Phys..

[17] Chebotareva A.B., Kost T.N., Stepanov A.S., Salazkin S.N., Shaposhnikova V.V. (2022). Aromatic cardo polyethers: Film adhesives for solar cell current collecting system. Russ. Chem. Bull..

[18] Chebotareva A.B., Untila G.G., Kost T.N., Stepanov A.S., Salazkin S.N., Shaposhnikova V.V. (2019). Bifacial silicon heterojunction solar cells with advanced Ag-free multi-wire metallization attached to ITO layers using new transparent conductive PAEK copolymers. Sol. Energy.

[19] Shaposhnikova V.V., Salazkin S.N., Donetskii K.I., Gorshkov G.V., Sharapov D.S., Mamedova I.A., Petrovskii P.V., Askadskii A.A., Bychko K.A., Kazantseva V.V. (2002). Synthesis and properties of cardo copoly(arylene ether ketones). Polym. Sci. Ser. A.

[20] Sakunts A.A. (1992). Effect of Synthesis Conditions on Molecular Weight Characteristics Aromatic Polyketone Based on 4,4’-difluorobenzophenone and Bisphenol. Ph.D. Thesis.

[21] Untila G.G., Kost T.N., Chebotareva A.B. (2020). ITO/SiOx/n-Si heterojunction solar cell with bifacial 16.6%/14.6% front/rear efficiency produced by ultrasonic spray pyrolysis: Effect of conditions of SiOx growth by wet-chemical oxidation. Solar Energy.

[22] Yusupov A.R., Gadiev R.M., Lachinov A.N., Kornilov V.M., Kalimullina L.R., Galiev A.F., Kian M., Salazkin S.N. (2021). Effect of polymer structure on the transport properties along the polymer/polymer interface. Synth. Metals.

[23] Mott N.F., Gurney R.W. (1940). Electronic Processes in Ionic Crystals.

[24] Mark P., Helfrich W. (1962). Space-Charge-Limited Currents in Organic Crystals. J. Appl. Physic.

[25] Tameev A.R., Lachinov A.N., Salikhov R.B., Bunakov A.A., Vannikov A.V. (2005). Mobility of Charge Carriers in Thin Poly(diphenylene phthalide) Films. Russ. J. Phys. Chem..

[26] Richardson O.W. (2003). Thermionic Emission from Hot Bodies.

[27] De Gennes P.G. (1979). Scaling Concepts in Polymer Physics.

[28] Xu Z., Park K.S., Diao Y. (2020). What is the assembly pathway of a conjugated polymer from solution to thin films?. Front. Chem..

[29] Yao Z.F., Wang J.Y., Pei J. (2022). Controlling morphology and microstructure of conjugated polymers via solution-state aggregation. Prog. Polym. Sci..

[30] Deng J., Zheng L., Ding C., Guo Y., Xie Y., Wang J., Ke Y., Li M., Li L., Janssenet R.A. (2023). Determinant Role of Solution-State Supramolecular Assembly in Molecular Orientation of Conjugated Polymer Films. Adv. Funct. Mater..

[31] Karamov D.D., Kornilov V.M., Lachinov A.N., Kraikin V.A., Ionova I.A. (2016). Atomic-force microscopy of submicron films of electroactive polymer. Tech. Phys..

[32] Karamov D.D., Il’yasov V.K., Lachinov A.N., Galiev A.F., Lachinov A.A. (2020). The Effect of Thickness of Submicron Films of Electroactive Polymers on Thermally Stimulated Depolarization Currents. Phys. Solid State.

[33] Zherebov A., Lachinov A.N., Genoe J., Tameev A.R. (2008). Polyheteroarylene films with intrinsic switching mechanism for nonvolatile memory applications. Appl. Phys. Lett..

